# miRNAs in bone tissue correlate to bone mineral density and circulating miRNAs are gender independent in osteoporotic patients

**DOI:** 10.1038/s41598-017-16113-x

**Published:** 2017-11-20

**Authors:** Sarah Kelch, Elizabeth R. Balmayor, Claudine Seeliger, Helen Vester, Jan S. Kirschke, Martijn van Griensven

**Affiliations:** 1 Experimental Trauma Surgery, Department of Trauma Surgery, Klinikum rechts der Isar, Technical University of Munich, Munich, Germany; 2 Department of Trauma Surgery, Klinikum rechts der Isar, Technical University of Munich, Munich, Germany; 3 Department of Neuroradiology, Klinikum rechts der Isar, Technical University of Munich, Munich, Germany

## Abstract

We previously demonstrated the aberrant expression of nine specific miRNAs in serum from osteoporotic patients. In the present study, we further evaluated the expression of these miRNAs in bone tissue, osteoblasts, and osteoclasts from 28 patients. We hypothesize that miRNA expression in serum from osteoporotic patients may be gender-independent. A further hypothesis is that the miRNA expression in bone could be correlated with BMD values. Moreover, intracellular expression of these osteoporosis-related miRNAs may indicate the role of these molecules during osteoporosis. Our results indeed show that miRNA expression in serum was gender-independent except for miR125b-5p. A correlation with BMD was confirmed for miR-21-5p, miR-24-3p, miR-93-5p, miR-100-5p and miR125b-5p with linear correlation coefficients r > 0.9. Intracellular studies revealed a simultaneous up-regulation of miR-21-5p, miR-93-5p, miR-100-5p and miR125b-5p in osteoblasts and in osteoclasts. miR-148a-3p up-regulation in cells was specific for osteoporotic osteoclasts. Altogether, miR-21-5p, miR-93-5p, miR-100-5p, and miR-125b-5p showed significant upregulation in serum, tissue and bone cells of osteoporotic patients. All except miR-125b-5p showed gender independent expression and good correlation to BMD values. Our results suggest that these miRNAs may be important for an earlier diagnosis of osteoporosis.

## Introduction

The World Health Organization (WHO) defines osteoporosis as bone mineral density (BMD) values of 2.5 standard deviations below the mean for young white adult women^1^. More recently, a consensus has been achieved in defining osteoporosis not solely based on BMD values, but also as a skeletal disorder characterized by impaired bone strength and quality with a subsequent increased risk of bone fractures^2^. Present estimates reveal that over 200 million people worldwide are affected by osteoporosis and the problem continues to grow^3^. The WHO named this disease a “silent epidemic of the 21^st^ century” resulting in more than 8.9 million osteoporotic fractures annually^1^. In fact, osteoporosis is often primarily diagnosed in patients that are admitted to the hospital for fracture treatment. Unfortunately, this diagnosis is mostly performed in a quite late phase of the disease. At that time, osteoporosis is usually already established or severe. The lifetime risk of osteoporotic fractures is relatively high. For example, the risk of hip fractures in osteoporotic patients is as high as 40%, thus in a comparable range as the lifetime risk of coronary heart disease^1^.

Currently, the diagnosis of osteoporosis is based solely on the determination of BMD by Dual energy X-ray absorptiometry (DXA). However, DXA-based diagnostics do not consider the contribution of factors like bone microarchitecture, cortical porosity or tissue and cellular level components. In addition, not all DXA measurements consider bone geometry. However, the DXA measurements based on hip axis length and neck-shaft angle do indeed take hip bone geometry into account. These factors have demonstrated an impact on osteoporosis^4^. Moreover, DXA measures an areal BMD, which is influenced by bone size; it cannot distinguish between cortical and trabecular bone. DXA is also a radiation-based diagnosis and it does not predict fracture risk and other complications^2^. It must be mentioned that the ability of DXA measurements to predict fracture risk remains somewhat controversial. For example, Marshall *et al*. concluded in a meta-analysis work including over 2000 fractures that measurements of BMD can predict fracture risk, but cannot identify individuals who will have a fracture^5^. A limitation more associated with therapy screening, is the fact that DXA measurements do not reveal the effect of several treatment drugs. Most of the patients taking bisphosphates as treatment for osteoporosis are recorded with similar DXA measurements before and after bisphosphonate treatment. This indicates that DXA determinations may not be used to follow treatment outcome.

Based on all the above-mentioned limitations, there is a critical need for earlier and more precise diagnostic measures of osteoporosis. Among several new techniques that are currently under development, the use of miRNAs as biomarkers for osteoporosis is attractive. miRNAs are small non-coding RNA segments that regulate many cellular biological functions. They can be found circulating in blood, but also in diverse tissues and organs and even at cellular levels. Particularly in the case of bone tissue, they are known to control bone homeostasis-related pathways^6^. miRNAs strongly contribute to the inhibition of important protein translations that are for instance needed to maintain healthy bone structures. To date, it is known that miRNAs play an important role in biological processes like cell division, apoptosis, differentiation and embryonic development^7^. miRNAs function by binding complementary sites of target mRNAs, thereby modulating the gene expression due to either translational inhibition or degradation of the mRNA. For example, miR-29 was found to regulate osteoblast activity by down-regulating osteonectin expression via the canonical Wnt pathway^8^. Similarly, Wang *et al*. demonstrated that elevated levels of miR-214 correlated with a lower degree of bone formation in bone tissue samples from aged patients^9^. The authors elegantly demonstrated that specific *in vitro* manipulation of miR-214 with an antagomir or mimic in osteoblasts resulted in a clear effect on osteogenesis of these cells. They identified ATF4 (activating transcription factor 4) as a target gene of miR-214.

Currently, several miRNAs have been identified to be up-regulated in serum and plasma of osteoporotic patients^10–12^. To a lesser extent, aberrant regulation of various miRNAs has been reported in bone tissue during osteoporosis^13,14^. Our group recently reported nine freely circulating miRNAs that are significantly up-regulated in the serum of osteoporotic patients^14^. Likewise, when bone tissue of the same osteoporotic patients was analyzed, five out of these nine miRNAs i.e. miR-21-5p, miR-23a-3p, miR-24-3p, miR-100-5p and miR-125b-5p were highly expressed^14^. The purpose of the present study was to more specifically investigate these osteoporosis-associated miRNAs for their correlation with BMD and gender as well as to examine their intracellular expression in bone-specific cells. Our goals were to *i)* determine the correlation of these miRNAs with clinical values such as BMD and gender and *ii)* study the intracellular expression of these miRNAs in different bone cell populations.

## Results

The miRNAs of interest analysed in our study were hsa-miR-21-5p, hsa-miR-23a-3p, hsa-miR-24-3p, hsa-miR-93-5p, hsa-miR-100-5p, hsa-miR-122-5p, hsa-miR-124-3p, hsa-miR-125b-5p, and hsa-miR-148a-3p. The accession number (www.mirbase.org) are tabulated in Table S1.

### All except one of the circulating miRNA are gender-independent in osteoporotic patients

**Figure 1:**
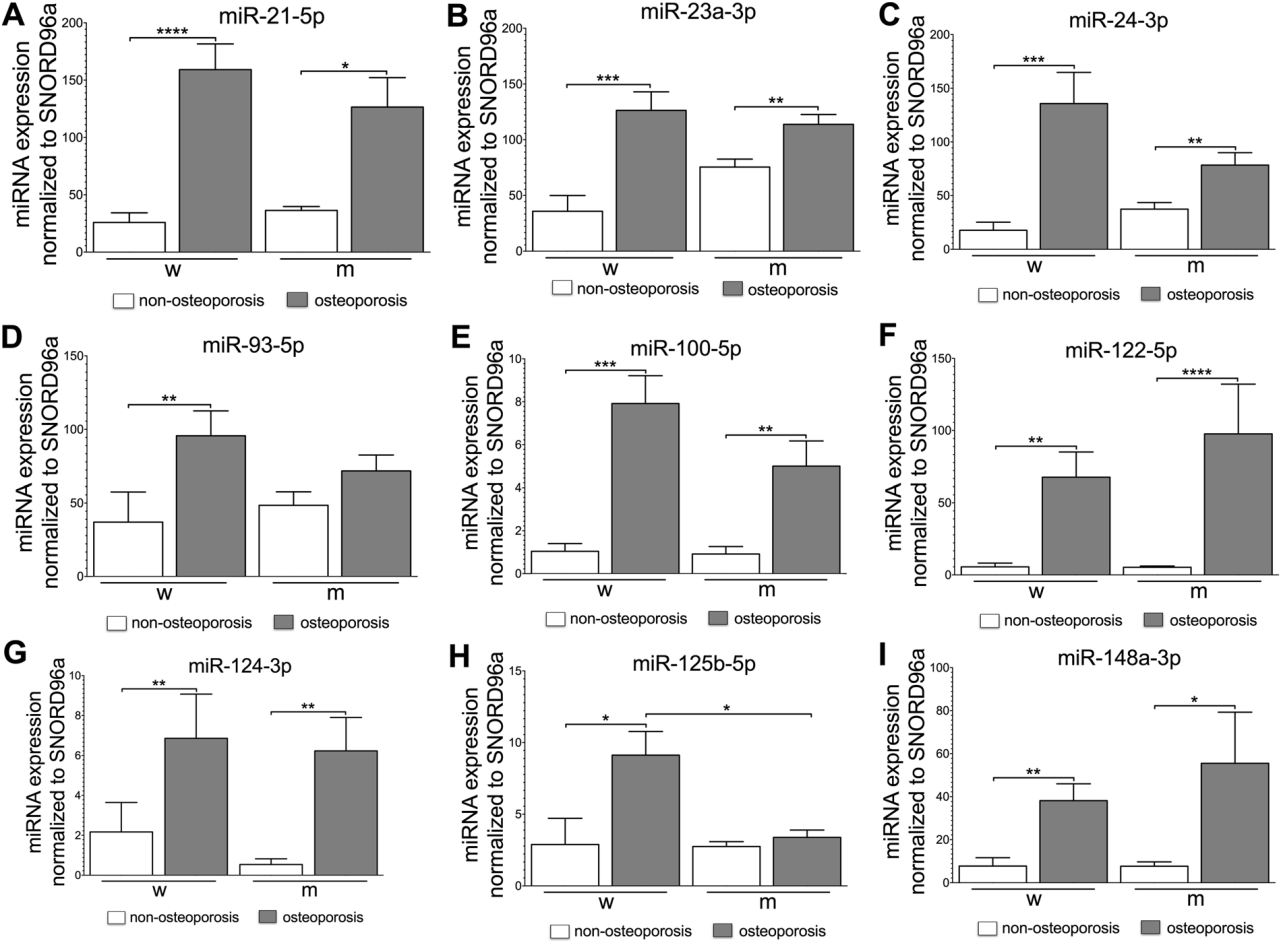
Relative expression of in serum circulating miRNA in male and female subjects with and without osteoporosis (each N = 7). The expression of miRNA-21-5p (**A**), miRNA-23a-3p (**B**), miRNA-24-3p (**C**), miRNA-93-5p (**D**), miRNA-100-5p (**E**), miRNA-122-5p (**F**), miRNA-124-3p (**G**), miRNA-125b-5p (**H**) and miRNA-148a-3p (**I**) are shown. Significant differences are indicated by *p < 0.05, **p < 0.01, ***p < 0.001, ****p < 0.0001. Normality of the data was tested by D’Agostino Pearson normality test. Statistical analysis was performed by means of a two-tailed Mann-Whitney test.

The expression level of all mature miRNAs determined in serum samples of all patients are shown in Fig. 1. Comparing the results obtained for female and male osteoporotic patients, no significant differences in miRNA relative expression were found for miR-21-5p (p = 0.745), miR-23a-3p (p = 0.723), miR-24-3p (p = 0.701), miR-93-5p (p = 0.630), miR-100-5p (p = 0.464), miR-122-5p (p = 0.06), miR-124-3p (p > 0.9) and miR-148a-3p (p = 0.648). In all cases, miRNAs were significantly up-regulated when compared to their non-osteoporotic counterparts (p < 0.03) with the exception of miR-93-5p (p = 0.12), which could not significantly distinguish between osteoporotic and non-osteoporotic males. miR-125b-5p was the only miRNA among the nine analyzed, which expression levels in serum showed to be gender-specific. This miRNA was significantly up-regulated in osteoporotic females (p = 0.013) and was almost at the same level comparing to non-osteoporotic and osteoporotic males (p = 0.675).

### miRNA expression levels in bone tissue correlate with BMD and distinguish between normal BMD, osteopenia and osteoporosis

**Figure 2:**
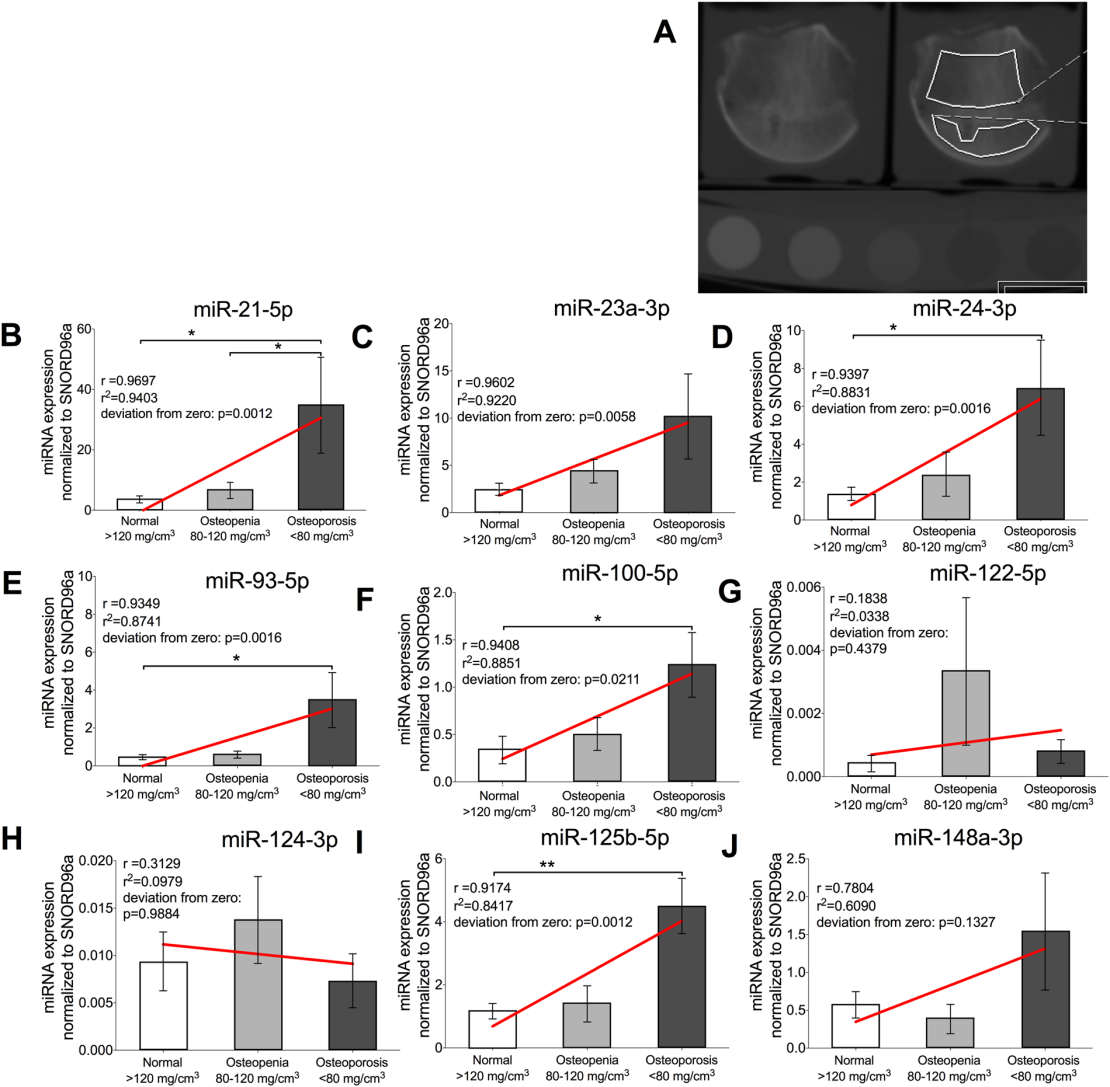
Relative miRNA expression and its correlation with BMD in bone tissue samples. Harvested femur heads (N = 28) were analyzed by qCT and the BMD was calculated (**A**). Column charts show miRNA relative expression for bone samples analyzed grouped by obtained BMD value (BMD ≥ 120 mg/cm^3^ normal, 120 mg/cm^3^ > BMD > 80 mg/cm^3^ osteopenic and BMD ≤ 80 mg/cm^3^ osteoporotic). The expression of miRNA-21-5p (**B**), miRNA-23a-3p (**C**), miRNA-24-3p (**D**), miRNA-93-5p (**E**), miRNA-100-5p (**F**), miRNA-122-5p (**G**), miRNA-124-3p (**H**), miRNA-125b-5p (**I**) and miRNA-148a-3p (**J**) are shown. Significant differences are indicated by *p < 0.05, **p < 0.01. Normality of the data was tested by D’Agostino Pearson test. Statistical analysis was performed by means of Kruskal-Wallis test corrected by Dunn’s test for multiple comparisons. Linear regression analysis was performed for miRNA relative expression vs. BMD and the resulting correlation coefficient (r) and coefficient of determination (r^2^) are shown for each graph.

miR-21-5p (p = 0.014), miR-24-3p (p = 0.022), miR-93-5p (p = 0.032), miR-100-5p (p = 0.04) and miR-125b-5p (p = 0.007) showed a significant up-regulation in bone tissue from osteoporotic patients when compared to the non-osteoporotic group (Fig. 2). Moreover, miRNA levels showed a linear correlation with the BMD values. For all these five miRNAs, the linear correlation coefficient r was found to be higer than 0.9 and presented an slope with a highly significant deviation from zero (p ≤ 0.001). In addition, the coefficient of determination r^2^ was in the range 0.84–0.94 indicating that more than 84–94% of the variance of the BMD is predictable by the miRNA expression. For miR-23a-3p, although its absolute expression in osteoporotic bone tissue was not significantly up-regulated (p = 0.102), it did show a linear correlation with the BMD values (r = 0.96 and r^2^ = 0.92, Fig. 2C). Remarkably, among the miRNAs, which expression correlated with BMD, miR-21-5p expression values additionally allowed to distinguish between osteopenia and osteoporosis (p = 0.048, Fig. 2B). miR-23a-3p, miR-122-5p, miR-124-3p and miR-148a-3p either did not show statistical significance when comparing non-osteoporotic and osteoporotic bones or their expression values did not correlate with BMD. Although a linear increase in miRNA levels for miR-23a-3p and miR-148a-3p was observed with increasing BMD values, no statistical significance was obtained between normal, osteopenia and osteoporosis (p > 0.1). Furthermore, for miR-148a-3p, the deviation from zero of the slope was not significant (p = 0.133, Fig. 2J). miR-122-5p and miR-124-3p showed no significant up-regulation in bone tissue of osteoporotic patients and no correlation with BMD values.

### miR-21-5p, miR-23a-3p, miR-24-3p, miR-93-5p, miR-100-5p and miR-125b-5p are up-regulated in osteoporotic osteoblasts

**Figure 3:**
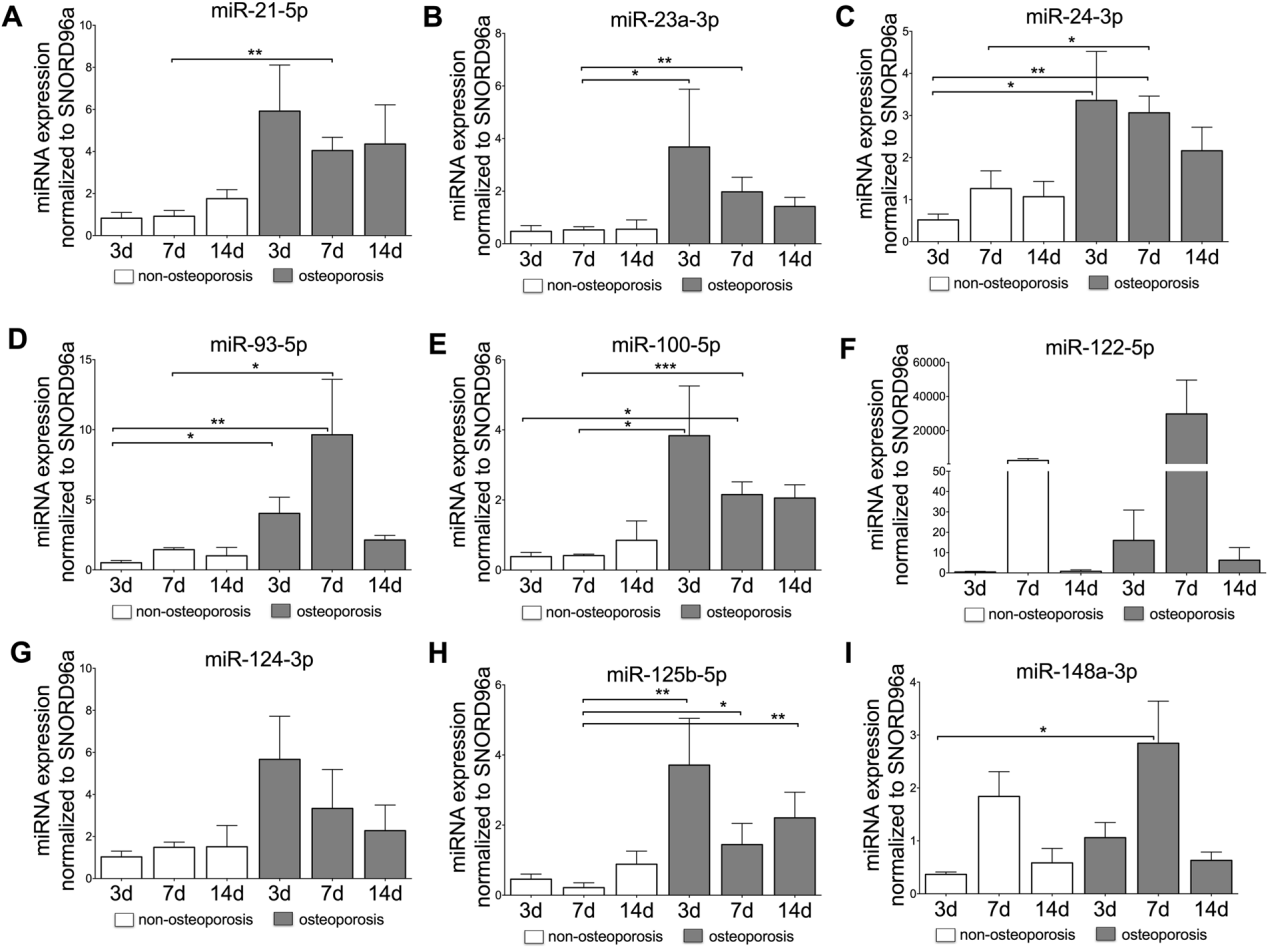
Relative expression of analyzed miRNAs in isolated osteoblasts. Osteoblasts were isolated from bone samples of osteoporotic and non-osteoporotic patients and cultured up to 14 days *in vitro*. The expression levels of miRNA-21-5p (**A**), miRNA-23a-3p (**B**), miRNA-24-3p (**C**), miRNA-93-5p (**D**), miRNA-100-5p (**E**), miRNA-122-5p (**F**), miRNA-124-3p (**G**), miRNA-125b-5p (**H**) and miRNA-148a-3p (**I**) are illustrated. Significant differences are indicated by *p < 0.05, **p < 0.01, ***p < 0.001. Normality of the data was tested by D’Agostino Pearson test. Statistical analysis was performed by means of Kruskal-Wallis test corrected by Dunn’s test for multiple comparisons.

A significant up-regulation in expression was obtained at day 3 for miR-24-3p (p = 0.048) and miR-93-5p (p = 0.039) (Fig. 3). After 7 days, significant up-regulations were found for miR-21-5p (p = 0.004), miR-23a-3p (p = 0.003), miR-24-3p (p = 0.03), miR-93-5p (p = 0.026), miR-100-5p (p < 0.001) and miR-125b-5p (p = 0.036). Interestingly, the expression levels in the osteoporotic osteoblasts show a tendency to decrease with time of culture (Fig. 3). Although this effect was not observed for all the miRNAs analysed. On the other hand, miR-122-5p (Fig. 3F), miR-124-3p (Fig. 3G) and miR-148a-3p (Fig. 3I) did not show a significant expression in osteoporotic osteoblasts when compared to their non-osteoporotic controls (p > 0.5). Nevertheless, the relative expression levels of these three miRNAs tended to be higher in osteoporotic cells when compared to non-osteoporotic osteoblasts for all times of culture analysed. Osteoporotic osteoblasts also showed significantly lower ALP activity when compared to non-osteoporotic cells (Figure S2, p < 0.0001 for 3 and 7 days of culture and p = 0.018). Interestingly, osteoporotic osteoblasts showed similar levels of mineralization as non-osteoporotic ones at early time points of evaluation (Figure S3, p > 0.05). Yet, at 14 days after culture (longest culture time evaluated), the osteoporotic cells did show a tendency to lower mineralization when compared to non-osteoporotic cells (Figure S3).

### miR-21-5p, miR-93-5p, miR-100-5p, miR-122-5p, miR-124-3p, miR-125b-5p and miR-148a-3p are up-regulated in osteoporotic osteoclasts

**Figure 4:**
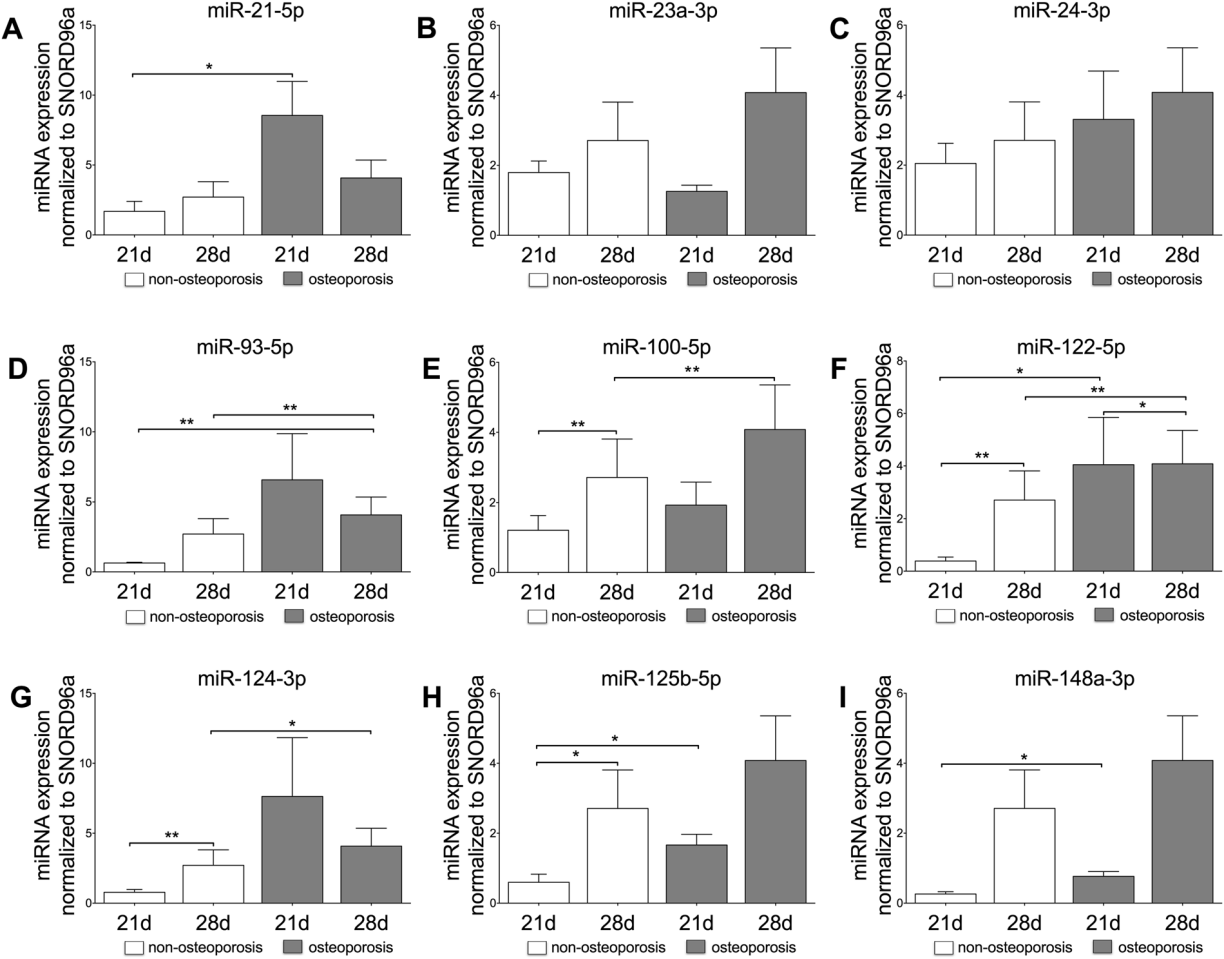
Relative expression of analyzed miRNAs in osteoclasts. Osteoclasts were generated from PBMCs isolated from blood samples of osteoporotic and non-osteoporotic patients. PBMCs were stimulated with M-CSF and RANKL for a period of 21 days or 28 days to generate the osteoclasts. The expression levels of miRNA-21-5p (**A**), miRNA-23a-3p (**B**), miRNA-24-3p (**C**), miRNA-93-5p (**D**), miRNA-100-5p (**E**), miRNA-122-5p (**F**), miRNA-124-3p (**G**), miRNA-125b-5p (**H**) and miRNA-148a-3p (**I**) are illustrated. Significant differences are indicated by *p < 0.05, **p < 0.01. Normality of the data was tested by D’Agostino Pearson test. Statistical analysis was performed by means of Kruskal-Wallis test corrected by Dunn’s test for multiple comparisons.

The expression levels of miR-21-5p, miR-122-5p, miR-125b-5p and miR-148a-3p were significantly up-regulated in osteoclasts of osteoporotic patients at day 21 when compared to non-osteoporotic ones (Fig. 4, p ≤ 0.048). This time point corresponds to the higher TRAP activity of osteoclasts generated from monocytes of osteoporotic patients (Figure S1). The TRAP activity of the osteoporotic osteoclasts was highly significantly increased in comparison with non-osteoporotic derived osteoclasts (p < 0.0001). Whether this increase is due to increased proliferation or differentiation could not be determined in this study. After 28 days of osteoclast differentiation, the expression levels of miR-125b-5p and miR-148a-3p further rised. This increase was, however, not significant when compared to the corresponding levels in osteoclasts from non-osteoporotic patients (Fig. 4H,I, p > 0.06). Other miRNAs, namely miR-93-5p, miR-100-5p and miR-124-3p gained a significant up-regulation after 28 days of culture in osteoporotic osteoclasts (Fig. 4D,E,G). Analyzing the overall time of osteoclast differentiation, miR-122-5p was the only miRNA that remained significantly up-regulated in osteoporotic osteoclasts during the entire culture time when comparing osteoporotic and non-osteoporotic cells (p = 0.019 for 21 days and p = 0.007 for 28 days). Finally, no significant up-regulation was found for miR-23a-3p and miR-24-3p in osteoporotic osteoclasts for any of the evaluated times of culture. Moreover, 21 days after monocyte differentiation to osteoclasts, only miR-23a-3p showed a down-regulation although not significant (p = 0.202, Fig. 4B).

## Discussion

Several studies have shown that specific abnormal regulation of miRNAs may correlate with the presence and progression of certain pathologies^15,16^. This is also the case of osteoporosis, for which miRNA profiles have been performed in serum and bone tissue^12–14^. Our previous work identified nine circulating miRNAs that were significantly up-regulated in 40 osteoporotic patients^14^. In the present study, we further investigated the expression of these miRNAs in serum samples of another set of osteoporotic patients with the specific aim to determine gender-dependent and BMD-dependent expression. Especially in the case of osteoporosis, differential variation between males and females may affect biomarker suitability. We found eight out of nine miRNAs to be gender-independent in serum samples from osteoporotic patients. Only miR-125b-5p displayed gender dependency and expression levels that were similar in osteoporotic and non-osteoporotic males. They were significantly lower compared to osteoporotic females. In other words, this miRNA could not be used to detect osteoporosis in male individuals, but only in females. Studying the current literature revealed very few studies that consider the effect of gender in circulating miRNA expression in other diseases or healthy volunteers. Most of these studies also concluded non-gender dependency for miRNA expression^17,18^. Freely circulating miRNAs in serum may be used as non-invasive biomarkers for diagnostic purposes. Thus, further investigations on the interconnection between their abnormal expression and disease progression is of crucial importance.

A further aim of our study was to evaluate local miRNA expression in bone tissue, osteoblasts and osteoclasts. In this case, our interest was directed towards the understanding of the contribution of the studied miRNAs in osteoporotic bone and bone cells rather than as using it as a diagnostic tool. Furthermore, by detecting abnormal expression of these miRNAs in osteoporotic osteoblasts and osteoclasts, possible targets could be identified for therapeutic inhibition. Detection of up-regulated miRNAs in bone tissue confirms our previously published data^14^. Moreover, miR-21-5p, miR-24-3p, miR-93-5p, miR-100-5p and miR-125b-5p were significantly correlated with BMD values. miR-21-5p could clearly distinguish non-osteoporotic, osteopenic and osteoporotic patients. This is in line with the positive correlation of serum miR-21 levels and BMD of hip and spine in normal, osteopenic and osteoporotic female patients^10^. Other attempts to correlate miRNA expression levels with BMD values have been done measuring miRNAs in circulating monocytes^19,20^ and whole blood^21^. We could not find any report in the literature on miRNA expression levels in bone tissue correlated to BMD values in osteoporosis. This may indicate a relation to disease progression in osteoporosis. Of note, none of our patients was treated for osteoporosis when included in the study. The possibility for an earlier diagnostis of osteoporosis or even disease prediction based on miRNA expression is desired. However, existing data is mostly based on patients in which osteoporosis is already settled and has been diagnosed by other clinical means. The clear limitation here is the possibility to clearly identify high-risk patients yet with a clinically negative osteoporosis diagnosis. Patients with dementia or a history of a prior fragility fracture may be considered as high-risk patients. Additionally, it may be practically difficult to evaluate the miRNA levels in the same individual before and after osteoporosis diagnosis. Our results on miR-21-5p are encouraging in this direction, as osteopenia (precursor to osteoporosis) could be diagnosed based on this miRNA expression levels. Thus, miR-21-5p may represent a potential candidate for an early osteoporosis diagnostic. Li *et al*. recently published data suggesting that miR-21 could be used as an early and highly sensitive biomarker for osteoporosis^10^. Interestingly, the authors found that miR-21 expression in serum was linked to osteoporosis and correlated good with clinically determined BMD. The authors found a significantly aberrant expression of miR-21 already in all osteopenic patients. Our results are in line with those presented by Li *et al*. and point to miR-21 as a potential candidate that merit further investigations.

**Figure 5:**
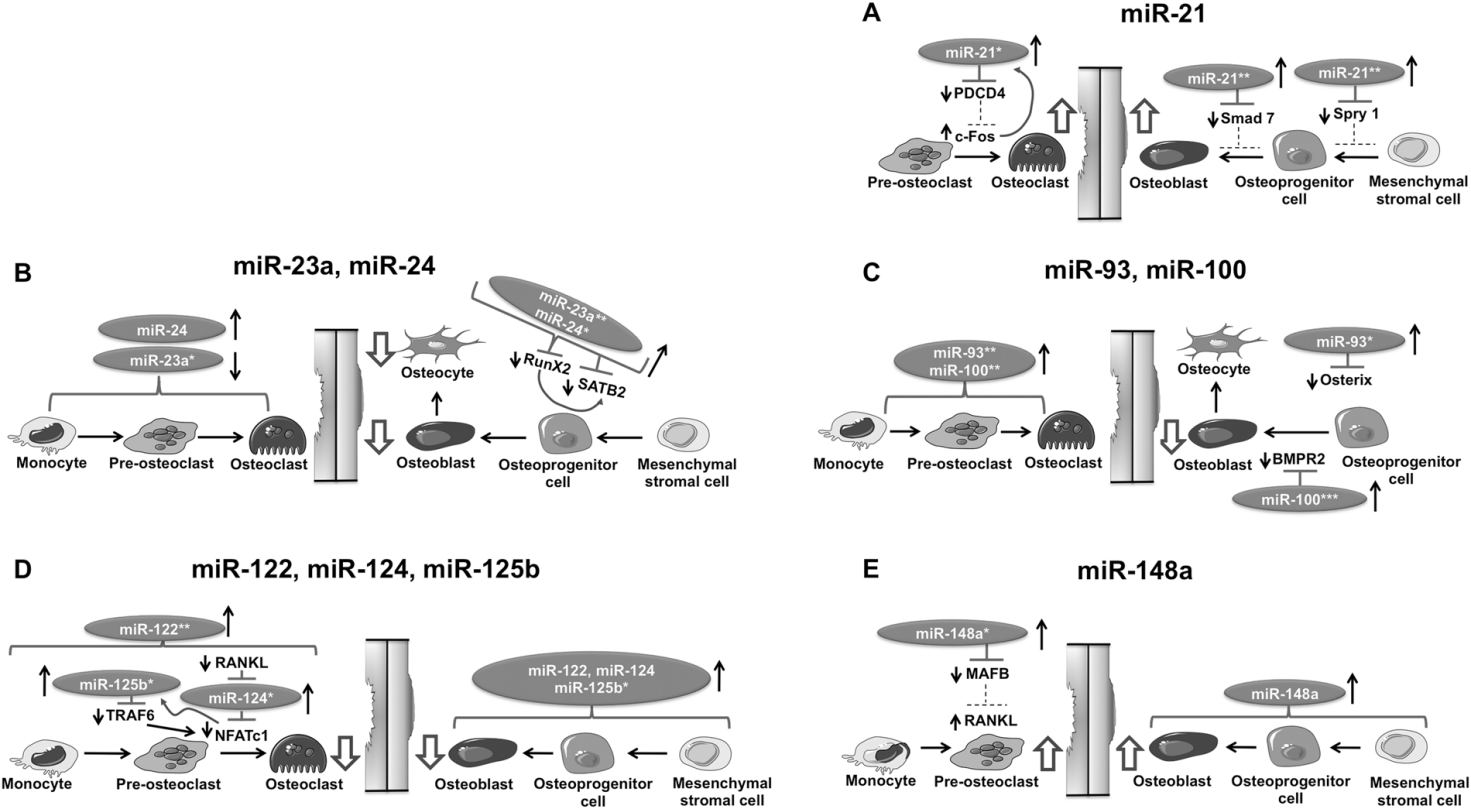
Schematic representation of the role of miRNA-21-5p (**A**), miRNA-23a-3p and miRNA-24-3p (**B**), miRNA-93-5p and miRNA-100-5p (**C**), miRNA-122-5p, miRNA-124-3p and miRNA-125b-5p (**D**) and miRNA-148a-3p (**E**) in bone remodeling during osteoporosis. For some of these miRNAs, the target(s) mRNA are known and they have been added to the schema. Solid lines in the diagram indicate direct action while dashed lines denote diminishing this action. Abbreviations used in the diagram are PDCD4: Programmed cell death protein 4, Smad7: Mothers against decapentaplegic homolog 7, Spry1: Protein sprouty homolog 1, RunX2: Runt-related transcription factor 2, SATB2: Special ATrich-sequence-binding protein 2, BMP-R2: Bone morphogenetic protein receptor type II, RANKL: Receptor activator of NF-κB ligand, TRAF6: Tumor necrosis receptor-associated factor 6, NFATc1: Nuclear factor of activated T-cells cytoplasmic 1 and MAFB: V-maf musculoaponeurotic fibrosarcoma oncogene homolog B.

Several of the up-regulated miRNAs during osteoporosis have been studied for their biological function in osteogenesis and/or osteoclastogenesis (reviewed in ref.^22^). Figure 5 schematically illustrates the reported roles of the miRNAs analysed in our study on osteoblast and osteoclast development. miR-21 has been extensively reported to facilitate osteoclastogenesis by a positive feedback loop that involves c-Fos/miR-21/PDCD4^23^. c-Fos up-regulates the expression of miR-21, which in turn down-regulates PDCD4. As a consequence, PDCD4-induced inhibition of c-Fos is repressed, which results in a higher generation of osteoclasts (Fig. 5A). This could explain the significant up-regulation of miR-21-5p in osteoporotic osteoclasts after 21 days of culture. In addition, the up-regulation of miR-21-5p found in female osteoporotic patients may be associated with low estrogen levels. Estrogen has been found to cause miR-21 down-regulation thereby resulting in osteoclast apoptosis^24^. Thus, miR-21 up-regulation in osteoporosis may mainly influence osteoclastogenesis. It is the balance that makes the difference, as some studies indicate an osteogenic effect of miR-21 *in vitro* ^25^.

miR-23a-3p and miR-24-3p are up-regulated in osteoporotic osteoblasts and are integrated in a miRNA cluster that is connected to RunX2 (runt-related transcription factor 2) and SATB2 (special AT-rich sequence-binding protein 2) regulating osteoblastogenesis (Fig. 5B). Literature studies indicate that RunX2 negatively regulates expression of the miRNA cluster 23a~27a~24, thereby increasing expression of bone markers and enhancing osteoblast differentiation^22,26^. In our osteoporotic scenario, the up-regulation of miR-23a-3p and miR-24-3p may lead to a down-regulation of RunX2, which in turn will result in lower formation of mature osteoblasts from osteoprogenitors and/or MSCs^27^.

miR-93-5p and miR-100-5p were shown to be significantly up-regulated in isolated osteoblasts and osteoclasts from osteoporotic participants. This may impair mineralization and maturation of osteoblasts via a miR-93/Osterix regulatory feedback loop^14,28^. miR-100 down-regulates the osteogenesis-promoting BMP-R2 (bone morphogenetic protein receptor type II)^14,29^. The effects of miR-93 and miR-100 on osteoclastogenesis have not being documented yet. One theory we consider plausible is that the regulation of miR-93 in osteoporotic osteoclasts may be related to high IL-8 levels. IL-8 stimulates osteoclastogenesis^30,31^; and the levels of this cytokine have been reported to be significantly elevated in osteoporotic patients^32,33^. Fabbri *et al*. demonstrated the presence of miR-93 consensus sequences in the 3′-UTR region of IL-8 mRNA, predicting a possible interaction of this cytokine with miR-93^34,35^.

miR-122-5p and miR-124-3p were found significantly up-regulated in serum and osteoclasts from osteoporotic patients^14,36^. It is known that miR-122 is a hepatic-specific miRNA that is expressed in patients with chronic liver diseases^37^. Osteoporosis is often found associated with chronic liver disease patients, with hormonal changes and vitamin D deficiency playing an important role^38^. However, none of the enrolled patient in our study presented a known diagnosis of liver diseases or vitamin D deficiency. miR-124, on the other hand, has been shown to regulate osteoclast differentiation, proliferation and migration via the suppression of NFATc1 (nuclear factor of activated T-cells cytoplasmic 1) expression^22^. Nakamachi *et al*. also reported the suppression of RANKL and NFATc1 by miR-124, thereby suppressing the differentiation of human osteoclasts as represented in Fig. 5D ^39^. miR-125b has also been associated with impaired osteoclastogenesis via a negative feedback loop with TRAF6 (tumor necrosis receptor-associated factor 6) and NFATc1^40^. In addition, a recent study reported by Cheng *et al*. showed a significant increase of miR-125b in bone marrow MSCs derived from osteoporotic patients^41^. A common fact for both, miR-124 and miR-125b, is that they also inhibit osteogenesis and thus have a negative impact in bone formation^22^. Thus, again, the balance between the effects on osteoclastogenesis and osteoblastogenesis is important. The net effect of these miRNAs in osteoporosis may still be the inhibition of osteoblastogenesis with concomitant impairment of bone formation.

We found miR-148a-3p up-regulated in osteoporotic osteoclasts. miR-148a is a pro-osteoclastic miRNA. Cheng *et al*. reported an up-regulation of miR-148a in osteoclasts differentiated from PBMCs via M-CSF/RANKL^42^. The authors also reported that miR-148a directly targeted MAFB mRNA (V-maf musculoaponeurotic fibrosarcoma oncogene homolog B) by binding to the 3′-UTR and repressed MAFB protein expression. *In vivo*, they could increase bone mass in ovariectomized mice by silencing of miR-148a using a specific antagomir.

A summary of our findings on miRNA expression using serum, bone tissue, osteoblasts and osteoclasts from osteoporotic individuals is presented in Table 2. In addition, our results on gender-dependency and BMD correlation have been also tabulated in Table 2 for a complete overview of the complex and interconnecting results.

**Table 2:** Overview of up-regulated miRNAs in serum, bone tissue, osteoblasts and osteoclasts (N = 28 samples evaluated for each sample type). Gender-dependency of circulating miRNA expression is indicated as well as the correlation between miRNA regulation in bone tissue and BMD (=bone mineral density) values. OB = osteoblast, OC = osteoclast

miRNA	Serum	Gender-dependent	Tissue	BMD correlation/Slope differs from zero	OBs	OCs
miR-21-5p		no		yes/yes		
miR-23a-3p		no		yes/yes		
miR-24-3p		no		yes/yes		
miR-93-5p		no		yes/yes		
miR-100-5p		no		yes/yes		
miR-122-5p		no		no		
miR-124-3p		no		no		
miR-125b-5p		yes		yes/yes		
miR-148a-3p		no		yes/no		

= Up-regulated,  = Significantly up-regulated.

A limitation of our study includes the low number of patients as well as the lack of control over dietary supplements (e.g. calcium, vitamin D) in the enrolled individuals. This may have interfered with our results. None of the patients were on active osteoporosis drugs such as bisphosphonates. In addition, the BMD values used for the correlation studies with miRNA levels represent the values obtained for the femoral heads only and may not be representative to an overall BMD. A further limitation is that patients with a fracture may have received some analgesic treatment that might have influenced miRNA expression. It is also not known how fractures influence the release of miRNAs. In our previous study, miRNA differences between patients with and without osteoporosis also differed in having a fracture or not^14^. Thus, unfortunately, we do not have evidence on the similarity of serum miRNA levels before and after fracture. Very little evidence has been published in this respect. One work from Weilner *et al*. recently explored the analysis of 175 miRNAs in serum samples of patients with recent osteoporotic fracture and aged-matched controls^12^. Significant regulation was found for miR-22-3p, miR-328-3p and let-7g-5p that was related to the osteoporotic fracture occurrence. But again, patients with fractures were compared to patients without fracture. Thus, the prediction of the regulation of miRNA in the same individual before and after the fracture would be practically very difficult to evaluate. This is because it is not known when the fracture is going to happen.

Our study presents innovative findings on miRNA regulation during osteoporosis on different levels of complexity (serum, tissue, cells). Specifically, the intracellular miRNA expression in osteoclasts has almost never been shown. Our results may allow us to identify miRNAs with high potential not only as biomarker for osteoporosis, but also as potential targets for therapeutic inhibition. The possibility of a non-gender specificity miRNA expression may be of great clinical advantage. On the other hand, by establishing a correlation between miRNA expression and BMD a multifactorial analysis may be performed that include different parameters of bone quality and strength, which may lead to higher accuracy of diagnosis. This may beneficially impact the way osteoporosis is diagnosed at present.

## Methods

### Ethics statement

The ethical committee of the Faculty of Medicine at the Technical University of Munich approved the experiments, collection of tissue and blood, and used protocols described inhere involving human material. The approval number is 2413/09a. In accordance with this approval, tissue and blood was collected with previous written patient informed consent and adhering to the newest guidelines of the declaration of Helsinki.

### Human sample collection

The patient demographic data are presented in Table 1. The recruited patients (N = 28) were admitted to our clinic with the diagnosis of hip fractures type AO 31-A/B (N = 14) or coxarthrosis Kellgren-Lawrence grade 3 or 4 (N = 14). The patients admitted with hip fractures were additionally diagnosed with osteoporosis based on radiographic or DXA examination. The patients with coxarthrosis were not having osteoarthritis (i.e. no signs of inflammation or presence of inflammatory mediators were detected. Further information on arthrosis and arthritis in ref.^43^). All enrolled patients (N = 28) were implanted with a total hip endoprosthesis and the femoral heads were collected for qCT, miRNA isolation and osteoblast isolation. Patients with known malignancy, inflammation, benign ovarian cysts except endometrioma, known chronic, systemic, metabolic and endocrine disease including polycystic ovarian syndrome, hormone therapy in the previous three months and any medical history of other inflammatory diseases were excluded from the study. Patients with estrogen replacement medication or vitamin D prescription were excluded.

**Table 1:** Demographical characteristics of all patients enrolled in the study.

	female osteoporotic patients	female non-osteoporotic patients
Included patients (N)	7	7
Age (y), mean (range)	81.9 (63–91)	74.2 (61–86)
Body mass index (kg/m^2^) ± SD	23.9 ± 1.9	25.6 ± 3
main diagnosis (beside osteoporosis)	femoral neck fracture	coxarthrosis
Volumetric BMD by qCT (mg/cm^3^) ± SD	67.8 ± 12.9	230.4 ± 33.4
	**male osteoporotic patients**	**male non-osteoporotic patients**
Included patients (N)	7	7
Age (y), mean (range)	78.0 (72–89)	68.6 (51–85)
Body mass index (kg/m^2^) ± SD	25.4 ± 4.2	28.0 ± 4.2
main diagnosis (beside osteoporosis)	femoral neck fracture	coxarthrosis
Volumetric BMD by qCT (mg/cm^3^) ± SD	32.0 ± 37.3	235.6 ± 39.7

No statistical differences concerning demographics in between the groups exist. Only the “volumetric BMD by qCT” values are significantly different between osteoporotic and non-osteoporotic patients (p < 0.05).

Following total hip endoprosthesis implantation, femoral heads were analysed via qCT (iCT, Philips, The Netherlands). A dedicated osteoporosis calibration phantom (Mindways, Austin, TX, USA) was used to perform the calculations of volumetric BMD. Patients were then classified according to the volumetric BMD values obtained for the bone specimens in: normal (>120 mg/cm^3^), osteopenic (80–120 mg/cm^3^) or osteoporotic (<80 mg/cm^3^)^44,45^.

Femoral head samples were harvested at most 8 hours after fracture occurrence (osteoporotic group) and within 30 minutes after removal from the body (both groups).

Blood samples were collected not only for serum miRNA determination, but also for isolation of monocytes to be differentiated into osteoclasts. Blood collection was done at most 2 hours post-fracture (osteoporotic group) or pre-operation (non-osteoporotic group).

### Primary human osteoblast isolation and culture

Cancellous bone from femoral heads was used for osteoblast isolations. Bone samples were mechanically shredded to small bone fragments by using a Luer forceps and transferred into a cell culture flask. Cells were allowed to grow out of the bone pieces into the culture plate^46^. A detailed osteoblast isolation protocol including materials used can be found in the supplementary material. In addition, employed characterization methods, i.e. ALP activity, alizarin red staining and quantification are also provided in the supplementary section.

Osteoblasts in passage 3 were plated at 1 × 10^4^ cells/cm^2^ and cultured for 3, 7 and 14 days. After each of these time points, cells were harvested and used for miRNA expression measurements.

### Primary human osteoclast generation and culture

Blood (40 ml) was collected in S-Monovette® EDTA K_3_ tubes (Sarstedt AG, Nümbrecht, Germany). Peripheral blood mononuclear cells (PBMCs) were isolated by using Lymphocyte Separation Medium (LSM, density 1077 kg/m^3^, Biowest, Nuaillé, France) following the manufacturer’s recommendations. Next, osteoclasts were generated by stimulating PBMCs with M-CSF (25 ng/ml) and RANKL (10 ng/ml later increased to 20 ng/ml). Detailed PBMCs isolation protocol as well as stimulation routine can be found in the supplementary material. Osteoclast phenotype was assessed by TRAP staining and activity. The description of the methods used can be found in the supplementary material.

Osteoclasts were then harvested either at 21 days or 28 days after PBMCs differentiation for miRNA expression and further analysis.

### miRNA extraction from serum

Osteoporotic patients (N = 14) and non-osteoporotic patients (N = 14) were subsequently divided according to their gender (i.e. N = 7 per group and gender).

#### Collection of blood samples

Blood was collected at most 2 hours post-fracture (osteoporotic group) or pre-operation (non-osteoporotic group). For this, 7.5 ml polypropylene tubes S-Monovette® (Sarstedt AG) were used. Blood samples were subsequently allowed to clot in the tubes by placing the tubes in an upright position for 30 minutes at room temperature. Next, the tubes were centrifuged at 1900g for 10 minutes. Clear serum supernatant was collected and transferred to sterile 2 mL Eppendorf tubes. One part of the sample was used for hemolysis test. For this, free hemoglobin was determined spectrophotometrically using a 3-points measurement at 415, 380 and 450 nm (Harboe method). Samples showing hemolysis were not included in further miRNA extraction steps. The rest of the samples were frozen and stored at −80 °C until further serum miRNA measurements.

#### miRNA extraction from serum samples

miRNA extraction was performed using miRNeasy Serum/Plasma Kit following the manufacturer’s recommendations (Qiagen). Detailed protocol is presented in the supplementary material. 200 µl serum was used per sample. In addition, 3.5 µl miRNeasy Spike-In control (1.6 × 10^8^ copies/µl *C. elegans* miR-39-3p miRNA mimic, Qiagen) was added just before RNA extraction. Obtained samples were stored at −80 °C until further cDNA transcription.

### miRNA extraction from bone tissue

Immediately after qCT scan, fresh bone samples from osteoporotic and non-osteoporotic patients were prepared for miRNA extraction.

#### Collection and preparation of bone samples

Attached tissue was removed from the bone samples using scalpels and sterile gauze. Cancellous bone tissue was harvested as a 5 × 20 mm cylindrical sample from the middle of each femoral head (Figure S4). Subsequently, the collected cylindrical samples were sectioned in small pieces by using a Luer forceps and washed twice with D-PBS. Thereafter, bone pieces were collected in TRI-Reagent (Sigma-Aldrich) and snap frozen in liquid nitrogen. Next, frozen bone samples were mechanically ground using a bench-top ball mill for wet/cryogenic grinding (MM 400, Retsch GmbH, Haan, Germany). Milling was performed at 30 Hz/sec for 40 seconds. Ground bone powder (0.5 ml) was collected with 1 ml of TRI-Reagent into a 2 ml Eppendorf tube and stored at −80 °C until further miRNA extraction.

#### miRNA extraction from bone tissue

miRNA isolation was performed using a phenol-chloroform extraction method. In brief, bone powder/TRI-Reagent mixtures were slowly thawed on ice. Next, 200 µl chloroform (PCR grade, Sigma-Aldrich) was added and the suspension was vortexed shortly. The mixture was incubated on ice for 10 minutes and subsequently centrifuged at 14000 g and 4 °C for 10 minutes. The upper and clear aqueous phase was transferred to a new Eppendorf tube and 500 µl isopropanol (molecular biology grade, Sigma-Aldrich) was added. After 10 minutes of incubation on ice, the samples were centrifuged at 14000 g, 4 °C for 10 minutes. The RNA pellet was washed several times with 70% ethanol. The ethanol was discarded and the tubes were allowed to air dry by placing them open for 5 minutes under the closed fume hood. The RNA pellet was suspended in 15 µl RNase-free water and stored at −80 °C until further cDNA transcription.

### miRNA extraction from isolated osteoblasts and generated osteoclasts

#### Collection of cellular material

After each of the indicated time points, cellular material was collected with 250 µl TRI-Reagent per well (48-well plates). Four wells corresponding to the same sample were pooled. Samples were stored at −80 °C until further RNA isolation.

#### miRNA extraction from cells

miRNA extraction was performed using the same phenol-chloroform extraction methodology described for bone samples. The initial volume of TRI-Reagent containing cells was of 1 ml. PCR grade chloroform (200 µl) was used for the extraction. Isolated RNA was stored at −80 °C until further cDNA transcription.

### Evaluation of the purity and integrity of isolated miRNA from serum, tissue and cells

Purity of isolated miRNA was determined spectrophotometrically using a BioPhotometer plus UV (Eppendorf AG, Hamburg, Germany). Values in the range 1.8–2.0 for A_260_/A_280_ and of 2.0–2.2 for A_260_/A_230_ were considered as pure RNA. Samples in which obtained values were out of the mentioned ranges were excluded from the evaluation. In addition, total RNA-integrity was assessed running aliquots of the isolated miRNA samples on a denaturing 1.5% agarose gel containing 7 µl ethidium bromide (EtBr). No degradation or additional by-products (unexpected bands) was detected. Further details on quality control of isolated miRNAs can be found in supplementary material.

### cDNA synthesis

Isolated miRNA from serum, tissue and cells was transcribed into complementary DNA (cDNA) using the miScript II RT Kit (Qiagen) according to the manufacturer’s instructions. A detailed procedure is provided in supplementary material. The RNA template (20 ng) was added to the reverse transcription master mix maintaining the final reaction volume in 20 µl for all the reactions. The mixture was gently mixed and incubated at 37 °C for 60 minutes followed by an incubation step at 95 °C for 5 minutes (Eppendorf mastercycler Nexus). cDNA was stored at −20 °C until further use in the qPCR reactions.

### miRNA qPCR quantification

The expression of miR-21-5p, miR-23a-3p, miR-24-3p, miR-93-5p, miR-100-5p, miR-122-5p, miR-124-3p, miR-125b-5p, and miR-148a-3p was analysed using the miScript SYBR Green PCR Kit (Qiagen). For qPCR, 3 µl cDNA template (10 ng/ml) per 25 µl reaction, containing: 12.5 µl 2x QuantiTect SYBR Green PCR Master Mix, 2.5 µl 10x miScript Universal Primer, 2.5 µl 10x miScript Primer Assay and 4.5 µl ultra-pure water. See supplementary material Table S1 for details on used miRNA primers, SNORD96a control primer and Spike-In primer. PCR amplification was conducted (CFX 96 PCR System, Bio-Rad, Munich, Germany) using the following settings: 95 °C for 15 minutes, 40 cycles of 94 °C for 15 seconds, 55 °C for 30 seconds and 70 °C for 30 seconds.

Efficiency of the complete qPCR process was confirmed by the Spike-In data. The percentage of recovery of the cel-miR-39-3p used as Spike-In control during the entire process was 94.4 ± 4.6%.

### Normalization of microRNA obtained data

The relative expression level of each miRNA (circulating miRNA as well as tissue/cellular specific) was normalized to the small RNA standard control molecule SNORD96a per sample (Normalized C_T_ = C_T_[target] − C_T_[SNORD96a]). As it is important to show also the real values of the non-osteoporotic patients, no normalization to this group was performed and results shown as 2^deltaCT^. The results are reported as miRNA expression normalized to the reference molecule SNORD96a.

### Statistical analysis

Results are given as column charts and depicted as mean ± SD. All data was analysed by D’Agostino-Pearson for normal distribution. Performed tests included two-tailed Mann-Whitney test, non-parametric Kruskal-Wallis test with Dunn’s correction for multiple comparisons, and t-test corrected for multiple comparisons using Holm-Sidak method. Linear regression analysis was performed for miRNA relative expression vs. BMD. All statistical analyses were performed using GraphPad Prism 6.0 (GraphPad Software, San Diego, USA). p < 0.05 was considered statistically significant.

### Data availability

The datasets generated during and/or analysed during the current study are available from the corresponding author on reasonable request.

## Electronic supplementary material

Supplementary Information
