# Supplementary material for: miRNAs in bone tissue correlate to bone mineral density and circulating miRNAs are gender independent in osteoporotic patients
Source: Sci Rep. 2017 Nov 20;7:15861. doi: 10.1038/s41598-017-16113-x (PMC5696459; doi:10.1038/s41598-017-16113-x)
Supplement: Supplementary file 1 — Supplementary Information [file 41598_2017_16113_MOESM1_ESM.pdf]

Electronic Supplementary Material for:

miRNAs in bone tissue correlate to bone mineral density and circulating miRNAs are gender independent in osteoporotic patients

Scientific Reports

Sarah Kelch[1,+], Elizabeth R Balmayor[1,+,*], Claudine Seeliger[1], Helen Vester[2], Jan S Kirschke[3], Martijn van Griensven[1]

[1]Experimental Trauma Surgery, Department of Trauma Surgery, Klinikum rechts der Isar, Technical University of Munich, Munich, Germany

[2]Department of Trauma Surgery, Klinikum rechts der Isar, Technical University of Munich, Munich, Germany

[3]Department of Neuroradiology, Klinikum rechts der Isar, Technical University of Munich, Munich, Germany

[+]these authors contributed equally to this work

*corresponding author:

Elizabeth Rosado Balmayor, PhD

Assistant Professor

Experimental Trauma Surgery, Department of Trauma Surgery,

Klinikum rechts der Isar, Technical University of Munich

Ismaninger Strasse 22

D-81675 Munich, Germany

Tel: +49-89-4140-9754; Fax: +49-89-4140-7526

Email: elizabeth.rosado-balmayor@tum.de

Supplementary Experimental Material

Materials

Cell culture medium (Dulbecco's Modified Eagle Medium (D-MEM) and alpha modification Minimum Essential Medium Eagle (α-MEM)), fetal calf serum (FCS), penicillin, streptomycin, HEPES, trypsin, Dulbecco's phosphate buffered saline (D-PBS) were purchased from Sigma-Aldrich (Munich, Germany). All materials and reagents used for miRNA expression analysis were obtained from Qiagen (Hilden, Germany). Macrophage colony-stimulating factor (M-CSF) and receptor activator of nuclear factor κB (RANKL) were purchased from PeproTech (Hamburg, Germany).

miRNAs of interest

In this study, we analysed the expression of hsa-miR-21-5p, hsa-miR-23a-3p, hsa-miR-24-3p, hsa-miR-93-5p, hsa-miR-100-5p, hsa-miR-122-5p, hsa-miR-124-3p, hsa-miR-125b-5p, and hsa-miR-148a-3p. The accession number for each miRNA (www.mirbase.org) has been tabulated in Table S1.

Methods

1. **Primary human osteoblast isolation**

Cancellous bone from femoral heads was used for osteoblast isolations. Bone samples were mechanically shredded to small bone fragments by using a Luer forceps and transferred into a cell culture flask. Culture medium (D-MEM with 10% FCS, 1% P/S and 50µg L-ascorbate-2-phosphate) was added and the flask was incubated for 7 days, with no further medium changes, at 37°C and 5% CO_2 in a humidified environment. Thereafter, the cell culture medium was changed to osteoblast expansion medium (Low glucose D-MEM, 5% FCS, 1% penicillin/streptomycin, 10 mM β-glycerol-phosphate disodium salt hydrate, 1.56 mM $CaCl_2$,

0.025 M HEPES, 100 nM dexamethasone and 0.2 mM L-ascorbic acid 2-phosphate) [49]. Cells were expanded until reaching passage 3 to ensure homogeneity of the culture and adequate purity. Cells in passage 3 were used for the miRNA expression measurements.

2. Characterization of isolated osteoblasts: Alkaline phosphatase activity and alizarin red as indication for calcium deposition

Alkaline phosphatase (ALP) activity was evaluated at days 3, 7 and 14 days after isolation. Thus, p-nitrophenyl phosphate (*p*NPP) was used as phosphatase substrate. In brief, isolated osteoblasts were washed twice with D-PBS and subsequently incubated with ALP substrate solution (i.e. 3.5 mM p-nitrophenyl phosphate in the presence of 50 mM glycine, 100 mM Tris-base and 2 mM magnesium chloride) for 30 minutes at 37°C. Next, 100 µl of the solution were transferred to a 96 well plate and the absorbance was measured at 405 nm using an Omega FLUOStar plate reader (BMG Labtech GmbH). The ALP activity was reported as means of p-nitrophenol content produced and calculations were performed based on a standard curve. Triplicates were evaluated in all cases.

Alizarin red staining was similarly performed at days 3, 7 and 14 days after osteoblast isolation to evaluate the presence of calcium deposits. In brief, ethanol-fixed cells were incubated with Alizarin red solution (5 mg/mL in D-PBS) for 10 minutes at room temperature. Stained cells were washed extensively with water and subsequently photographed using a microscope (BZ-9000, Keyence). Calcium deposits are stained as red spots. Thereafter, the alizarin red dye was extracted with 100 mM cetylpyridinium chloride by incubating the stained cells for 3 hours at room temperature. Absorbance was measured at 570 nm and calculations were performed based on a standard curve. Experiments were performed in triplicates.

3. Primary human osteoclast generation

Peripheral blood mononuclear cells (PBMCs) were isolated from collected blood by using Lymphocyte Separation Medium (LSM, density 1077 kg/m^3, Biowest, Nuaillé, France). In brief, 30 ml of the collected patient's blood was carefully layered on top of 20 ml LSM. Subsequently, the tubes were centrifuged during 20 minutes at 1000g without brakes. PBMCs were then collected from the interphase layer. Washed PBMCs were resuspended in α-MEM supplemented with 10% FCS and 1% penicillin/streptomycin (α-MEM complete) and plated at 3 x 10^6 cells/cm^2. 24 hours later, the cell culture medium was changed to α-MEM complete additionally containing 25 ng/ml M-CSF. After 6 days of M-CSF stimulation, 10 ng/ml RANKL were added and the cells were cultured under these conditions for the next 24 hours. At that time, the cell culture medium was replaced with α-MEM complete additionally containing solely 20 ng/ml RANKL. Osteoclasts were then harvested either at 21 days or 28 days after culture for further analysis.

4. Characterization of functional osteoclasts generated from PBMCs: TRAP staining and activity

In order to assess osteoclast differentiation and function, tartrate-resistant acid phosphatase (TRAP) activity was determined. TRAP staining was performed to visualize the presence of osteoclasts. Thus, confirming successful differentiation from PBMCs. TRAP activity was examined to determine the level of osteoclast resorption activity.

The TRAP staining was determined at 21 and 28 days after initiation of osteoclast differentiation. In brief, cells were fixed with 4% formaldehyde for 5 minutes and subsequently allowed to air-dry at room temperature. Next, cells were covered with a TRAP staining solution (i.e. 40 mM sodium acetate, 10 mM sodium tartrate, 0.01% naphthol AS-MX phosphate, 0.06% fast red violet LB salt, and 1% N,N-dimethylformamide) and incubated for 20 minutes at 37°C. After a washing step with Dulbecco's phosphate buffered

saline (D-PBS), photographs were taken using the fluorescence microscope (BZ-9000, Keyence, Osaka, Japan). Experiments were performed in triplicates.

The TRAP activity of stimulated PBMCs was determined at 6, 21 and 28 days after initiation of osteoclast differentiation. For this analysis, 50 µl of cell culture supernatant were mixed with 150 µl 5 mM 4-nitrophenyl phosphate disodium solution (pNPP) in a 96 well plate. After incubation for 1 hour at 37°C, the reaction was stopped by addition of 50 µl 3 M NaOH and the absorbance was determined at 405 nm using an Omega FLUOStar plate reader (BMG Labtech GmbH, Jena, Germany). Experiments were performed in triplicates. TRAP activity was calculated based on a standard curve.

5. miRNA extraction from serum samples using miRNeasy Serum/Plasma Kit

Serum samples were thawed on ice. Next, 1000 µl QIAzol lysis reagent was added to 200 µl serum sample and vortexed to ensure complete mixing. The mixture was incubated at room temperature for 5 minutes and 3.5 µl miRNeasy Spike-In control (1.6 x 10^8 copies/µl *C. elegans* miR-39-3p miRNA mimic, Qiagen) was subsequently added. RNA extraction was performed by adding 200 µl chloroform to the mixture. After a brief vortex, the mixture was incubated at room temperature for 2 minutes and subsequently centrifuged at 12000g, 4°C for 15 minutes. The upper aqueous phase was thoroughly mixed with 100% ethanol and the mixture was pipetted into an RNeasy MinElute spin column (Qiagen). After a short centrifugation (15 seconds) at 8000g, 700 µl buffer RWT and 500 µl buffer RPE were added to the spin columns always followed by a short centrifugation step (8000g). Finally, the spin columns were washed with 80% ethanol and the RNA was eluted by adding 15 µl RNase-free water and centrifuging for 1 minute at full speed. Obtained samples were stored at -80°C until further cDNA transcription.

6. Evaluation of the purity and integrity of isolated miRNA from serum, tissue and cells

To guarantee a correct quantification of circulating miRNAs levels as well as miRNAs in tissue and cellular material, several quality control steps were applied namely, hemolysis test to serum samples, use of RNA spike-in control and RNA integrity checks.

- Hemolysis test to serum samples: Free hemoglobin was determined spectrophotometrically using a 3-points measurement at 415, 380 and 450 nm (Harboe method). Samples showing hemolysis were not included in further miRNA extraction steps.
- RNA spike-in control: *C. elegans* miR-39-3p miRNA mimic (Qiagen).
- RNA integrity checks: *Purity of isolated miRNA* was determined spectrophotometrically (BioPhotometer plus UV, Eppendorf AG) by reading the absorbance at 230, 260 and 280 nm. The ratio A_{260}/A_{280} was considered as indication of the presence of contaminants. Values in the range of 1.8-2.0 were considered as pure RNA. In addition, the ratio A_{260}/A_{230} was used as secondary measurement of RNA purity. In this case, a range of 2.0-2.2 was considered as pure RNA. Samples in which obtained A_{260}/A_{280} and A_{260}/A_{230} values were out of the mentioned range were excluded from the evaluation.

  In addition, *total RNA-integrity* was assessed running aliquots of the isolated miRNA samples on a denaturing 1.5% agarose gel containing 7 µl ethidium bromide (EtBr). Of each sample, 0.3 µg miRNA was brought into solution with 15 µl RNase-free water and 5 µl loading buffer. The samples were placed in the gel. Subsequently, 90 volts were applied for 40 minutes. The gels were evaluated using INTAS Gel iX20 Doc System (Intas Science Imaging Instruments GmbH, Göttingen, Germany).

7. cDNA synthesis using the miScript II RT Kit (Qiagen)

A reverse transcription master mix was prepared by mixing together 4 µl 5x miScript HiSpec Buffer, 2 µl 10x miScript Nucleics Mix (contains dNTPs, rATP, oligo-dT primers, and miRNA reverse transcription internal control), 2 µl miScript Reverse Transcriptase Mix and adequate amount of RNase-free water. By using miScript HiSpec Buffer, only mature miRNAs are converted into cDNA. RNA samples (i.e. isolated miRNA from serum, tissue and cells) were thawed on ice and 20 ng RNA were used as template in the reverse transcription reaction. The RNA template was added to the reverse transcription master mix maintaining the final reaction volume in 20 µl for all the reactions. The mixture was gently mixed and incubated at 37°C for 60 minutes followed by an incubation step at 95°C for 5 minutes in an Eppendorf mastercycler Nexus. Undiluted cDNA was stored at -20°C until further use in the qPCR reactions.

Supplementary Table S1. Details of used assay primers during qPCR evaluations. SNORD96A = small nucleolar RNA was used as small RNA standard control molecule and Ce_miR-39_1 as Spike-In primer.

**Target miRNA**	**Accession # mirbase.org**	**Assay Primer**	**Catalogue # Qiagen**	**Primer Sequence**
hsa-miR-21-5p	MIMAT0000076	Hs_miR-21_2	MS00009079	5'UAGCUUAUCAGACUGAUGUUGA3'
hsa-miR-23a-3p	MIMAT0000078	Hs_miR-23a_2	MS00031633	5'AUCACAUUGCCAGGGAUUUCC3'
hsa-miR-24-3p	MIMAT0000080	Hs_miR-24_1	MS00006552	5'UGGCUCAGUUCAGCAGGAACAG3'
hsa-miR-93-5p	MIMAT0000093	Hs_miR-93_1	MS00003346	5'CAAAGUGCUGUUCGUGCAGGUAG3'
hsa-miR-100-5p	MIMAT0000098	Hs_miR-100_1	MS00003388	5'AACCCGUAGAUCCGAACUUGUG3'
hsa-miR-122-5p	MIMAT0000421	Hs_miR-122a_1	MS00003416	5'UGGAGUGUGACAAUGGUGUUUG3'
hsa-miR-124-3p	MIMAT0000422	Hs_miR-124a_1	MS00006622	5'UAAGGCACGCGGUGAAUGCC3'
hsa-miR-125b-5p	MIMAT0000423	Hs_miR-125b_1	MS00006629	5'UCCCUGAGACCCUAACUUGUGA3'
hsa-miR-148a-3p	MIMAT0000243	Hs_miR-148a_1	MS00003556	5'UCAGUGCACUACAGAACUUUGU3'
SNORD96A	-	Hs_SNORD96A_11	MS00033733	5'UAGCUUAUCAGACUGAUGUUGA3'
cel-miR-39-3p	MIMAT0000010	Ce_miR-39_1	MS00019789	5'UCACCGGGUGUAAAUCAGCUUG3'

Supplementary Figures

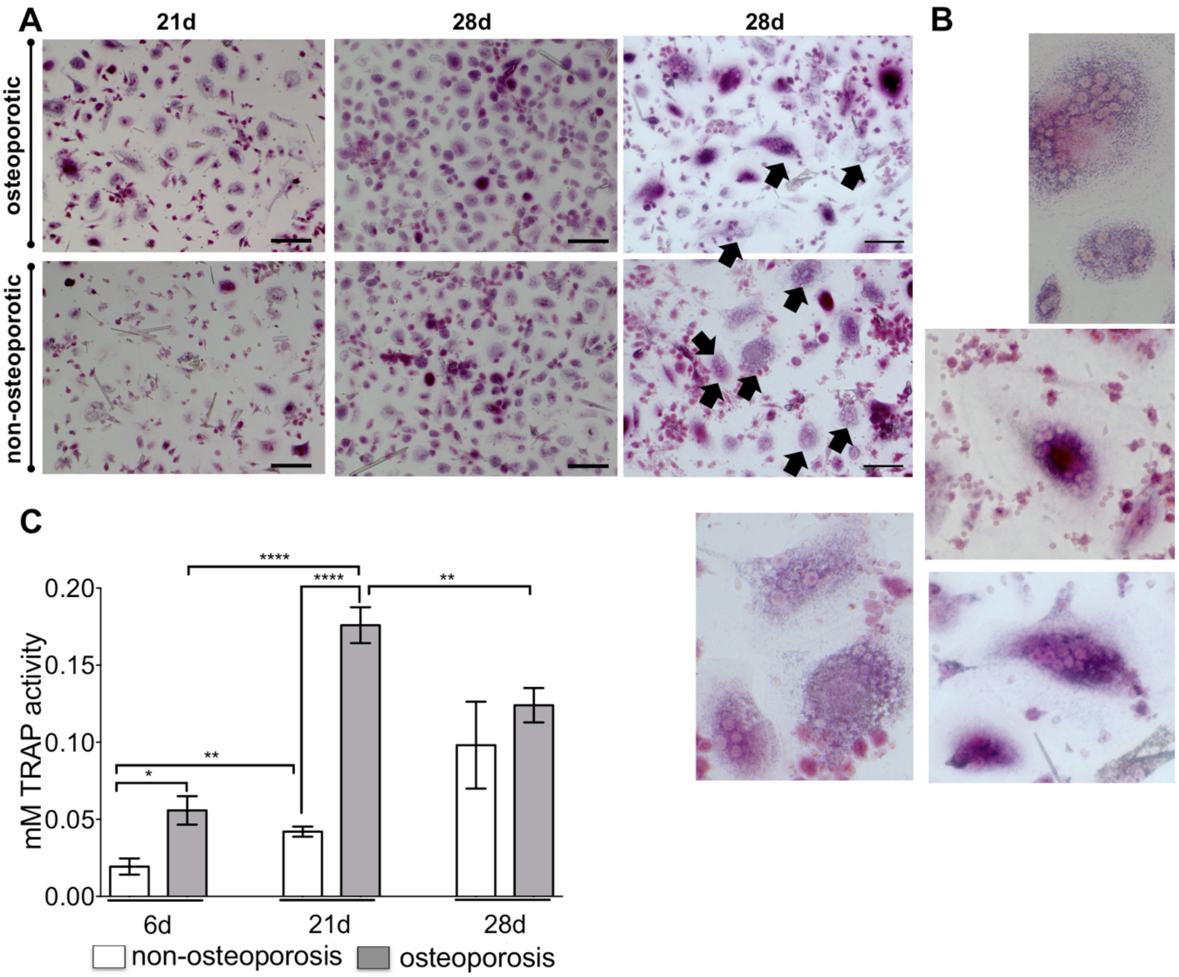

Supplementary Fig. S1. TRAP staining (A, B) and activity (C) of generated osteoclasts after respective periods of M-CSF and RANKL stimulation. Black arrows in (A) indicate multi-nucleated cells. In addition, higher magnification images provided in (B) illustrate the multinucleated character of isolated osteoclasts. Significant differences are indicated in (C) by $*p < 0.05$, $**p < 0.01$, $***p < 0.001$, $****p < 0.0001$. Normality of the data was tested by D'Agostino Pearson test. Statistical analysis was performed by means of a multiple t-test corrected for multiple comparisons using Holm-Sidak. Scale bar = 100 µm.

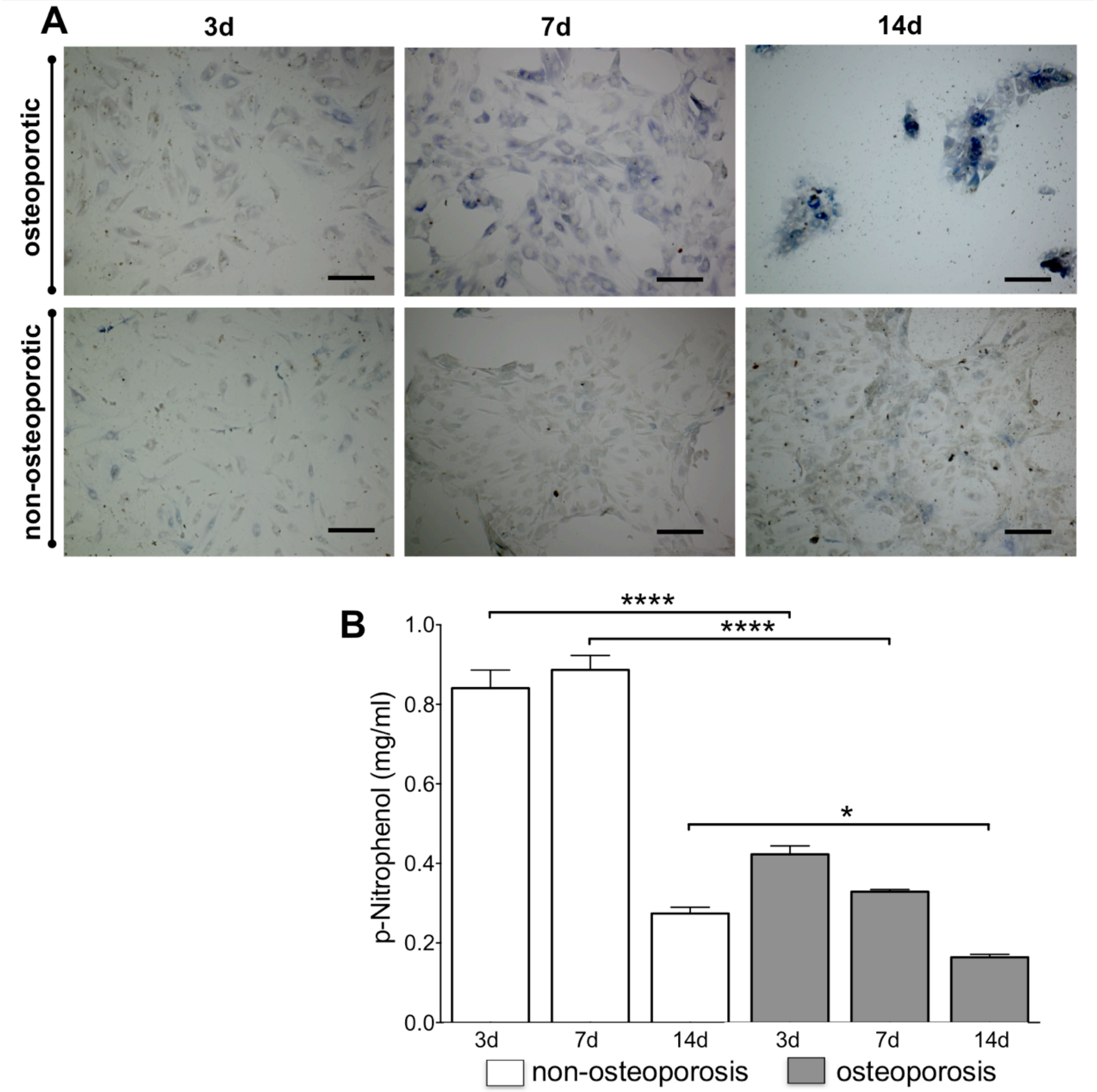

Supplementary Fig. S2. ALP staining (A) and activity (B) of isolated osteoblasts after 3, 7 and 14 days of culture. Significant differences are indicated by *$p < 0.05$ and ****$p < 0.0001$. Normality of the data was tested by D'Agostino Pearson test. Statistical analysis was performed by means of One-way ANOVA. Scale bar = 200 μm.

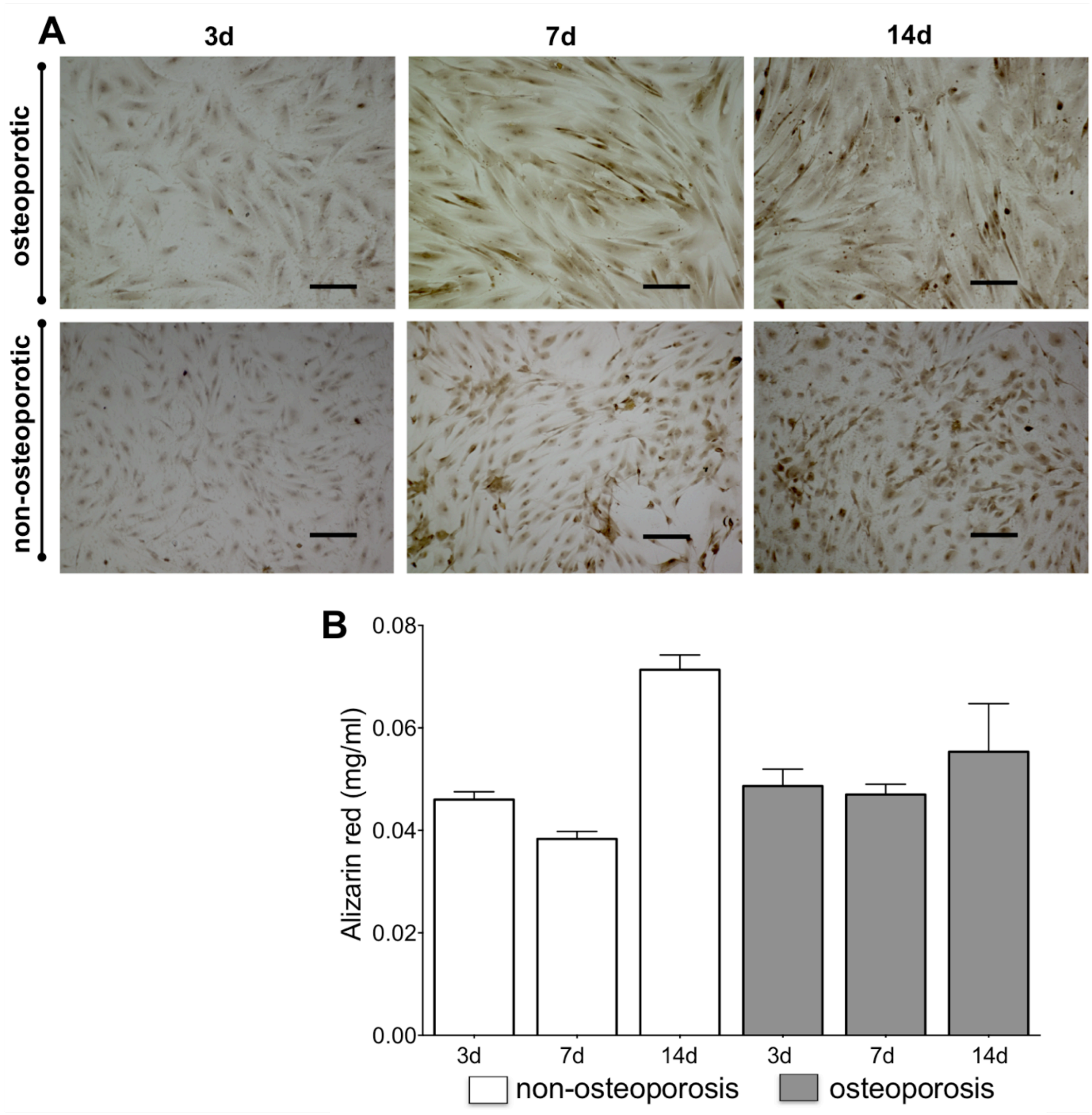

Supplementary Fig. S3. Alizarin red staining (A) and quantification (B) of isolated osteoblasts after 3, 7 and 14 days of culture. No significant differences were obtained. Normality of the data was tested by D'Agostino Pearson test. Statistical analysis was performed by means of One-way ANOVA. Scale bar = 200 μm.

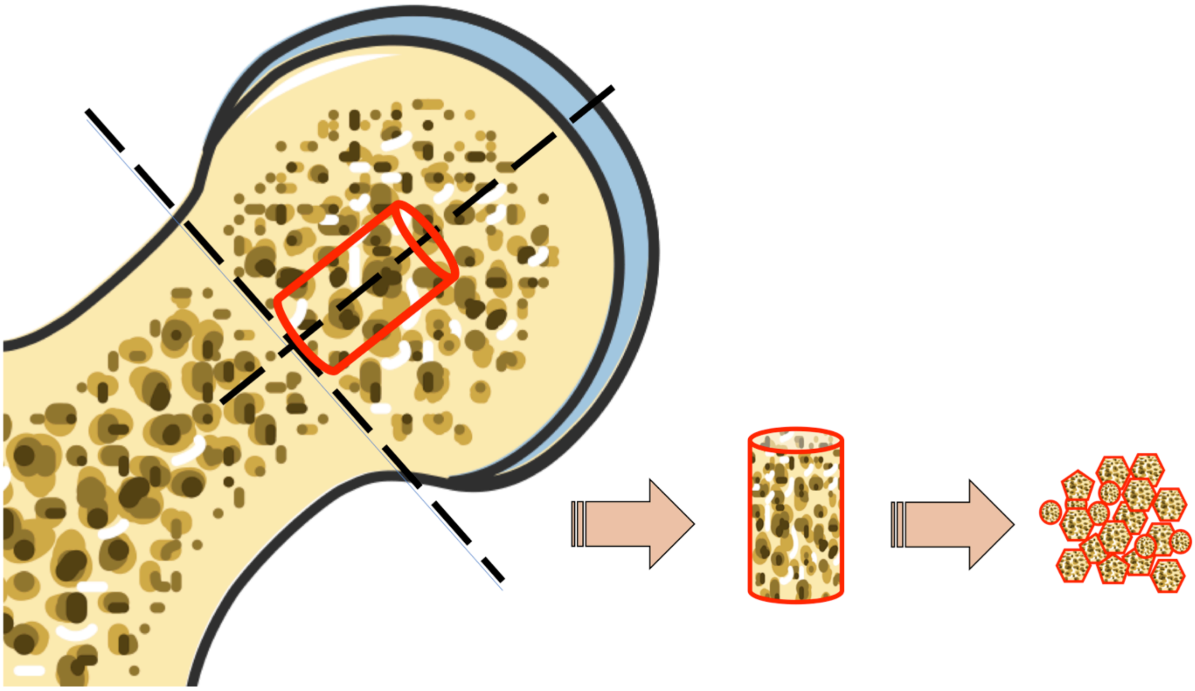

Supplementary Fig. S4. Schematic representation of the standardized measurement site of miRNA expression in bone tissue. The bone tissue used for miRNA qPCR analysis corresponded in all samples to the cancellous bone tissue of an approximately 5x20 mm cylindrical sample harvested in the middle of each femoral head. Subsequently, the collected cylindrical sample was sectioned in small pieces using a Luer.
